# MARGINAL NOTES, March 2025. Belonging

**DOI:** 10.1099/jmm.0.002001

**Published:** 2025-03-31

**Authors:** Timothy J. J. Inglis

**Affiliations:** 1 Schools of Medicine and Biomedical Sciences, University of Western Australia, Crawley, 6009, Australia

**Keywords:** biodefence, biomedical publishing, clinical microbiology, emerging infectious diseases, journal editors, sepsis and bloodstream infection

2025 is a transition year, when our senior editorial team will change over in a process designed to preserve business continuity. We have already welcomed a new production editor, Emily Arnold, into the role and now switch in a new Editor-in-Chief and Deputy Editor-in-Chief.

It would be difficult to match the disruption caused by the pandemic, and maybe finding an Editor-in-Chief in one of the safest locations on Earth was a nod to sustainability in pandemic times. Learning how to deal with the paradox of staying together while maintaining physical distance gave us a better sense of common purpose, leading us to revise our statement of scope. The long-form document describing our scope was the product of many conversations [1], laying out an inclusive vision for the journal that represents the broad medical science community we have become. That contribution towards a cohesive community of practice is a reference point for the incoming leadership team standing up for evidence-based biomedical science, as mainstream science comes under political attack. The patient, methodical explanation of natural phenomena through widely accepted investigational methods is too unexciting for unchecked social media sounding boards. Nevertheless, a strong grasp of scientific method is needed among the load-bearing timbers of evidence-based medicine. An emerging battleground where we need to act on our professional convictions to establish standards for trustworthy knowledge is the current debate about the use of artificial intelligence in biomedical publishing. We can’t let the grain of truth slip through our fingers among the shifting sands of populist opinion.

While our statement of scope is necessarily broad, our special collections reflect emerging areas where our community of practice concentrates its efforts. I have chosen three themes as stretch goals: sepsis and bloodstream infection, biodefence and applied machine learning. Submissions on these themes are strongly encouraged. Of course, antimicrobial resistance intersects with all three and will likely consume our efforts for years to come. The emerging infectious disease consequences of climate change, such as those occurring locally in the wake of tropical cyclone Alfred, also raise major public health issues. Whether due to weather effects, breakdown of the supply chain or regional hostilities, sustaining clinical public health microbiology is a persistent challenge. Innovation, repurposing and logistic considerations are undervalued perspectives on our daily work. For example, during the dark days of World War II, Britain lost its main supply of bacteriological agar and had to find an alternative from seaweed growing around its coastline. The stay calm and carry on theme seems appropriate for our troubled times.

As I come to the end of my tenure and hand over responsibility for the Journal to Drs Tina Joshi and Matthew Diggle, I'm confident they will continue to foster a sense of belonging among our community of practice.

Tim Inglis, Western Australia.
